# P-352. Resistance of the Public Health Threat Emerging Pathogen Candida Auris from Multiple Sources to Antifungal Agents (ARIA 2020-2022)

**DOI:** 10.1093/ofid/ofae631.553

**Published:** 2025-01-29

**Authors:** Stephen Hawser, Federica Monti, Sara Olari, Nimmi Kothari

**Affiliations:** IHMA Europe, Monthey, Valais, Switzerland; IHMA, Monthey, Valais, Switzerland; IHMA, Monthey, Valais, Switzerland; IHMA, Monthey, Valais, Switzerland

## Abstract

**Background:**

As highlighted by the Centers for Disease Control (CDC), resistance to antifungal therapies leads to high morbidity and moderate to severely high mortality. In recent years, infections caused by *Candida auris* have been extremely difficult to treat and *C. auris* is now labelled as a public health threat. ARIA is a global surveillance initiative collecting yeast and fungal isolates from worldwide sources designed to determine resistance to antifungal agents and trends over time. The current study reports *C. auris* isolates from the ARIA project and outlines the susceptibility of these isolates to clinically used antifungal agents.

**Figure:**
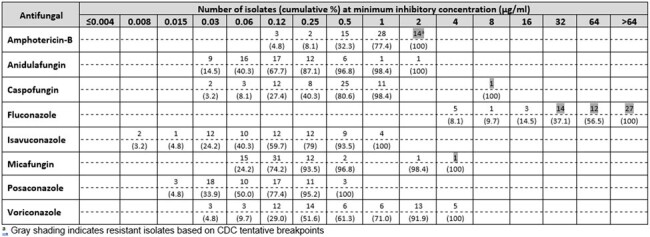

**Methods:**

A total of 62 clinical isolates of *C. auris* were collected between 2020 and 2022. These originated from specific hospital sites in Germany (1), India (26), Kuwait (5), Panama (20) and the United Arab Emirates (10). All isolates were non-consecutive non-duplicate isolates. Isolates were identified via MALDI-TOF mass spectroscopy and susceptibility testing was performed in line with Clinical Laboratory Standard Institute (CLSI) guidelines using amphotericin B, anidulafungin, caspofungin, fluconazole, isavuconazole, micafungin, posaconazole and voriconazole. As there are no accepted breakpoints for *C. auris*, CDC tentative e breakpoints were adopted.

**Results:**

MIC distributions with respect to CDC tentative breakpoints specific for *C. auris* are shown in the Table. 14 of the 62 isolates (23%) were resistant to amphotericin B, 1 isolate (1.6%) resistant to caspofungin, 53 isolates (85%) resistant to fluconazole and 1 isolate resistant to micafungin. Notably, all amphotericin-B resistant isolates were also fluconazole-resistant.

**Conclusion:**

The data from the ARIA study further emphasize the threat posed by *C. auris* considering its ability to resist commonly used antifungal agents. Continued monitoring of *C. auris* susceptibility is essential.

**Disclosures:**

**All Authors**: No reported disclosures

